# Early Adolescent Skills for Emotions (EASE) intervention for the treatment of psychological distress in adolescents: study protocol for randomised controlled trials in Lebanon and Jordan

**DOI:** 10.1186/s13063-019-3654-3

**Published:** 2019-09-02

**Authors:** Felicity L. Brown, Frederik Steen, Karine Taha, May Aoun, Richard A. Bryant, Mark J. D. Jordans, Aiysha Malik, Mark van Ommeren, Adnan Abualhaija, Ibrahim Said Aqel, Maha Ghatasheh, Rand Habashneh, Marit Sijbrandij, Rabih El Chammay, Sarah Watts, Aemal Akhtar, on behalf of the STRENGTHS-consortium

**Affiliations:** 1 Research and Development Department, War Child Holland, Amsterdam, The Netherlands; 2 War Child Holland Lebanon Office, Beirut, Lebanon; 3 School of Psychology, University of New South Wales, Sydney, Australia; 4 Amsterdam Institute of Social Science Research, University of Amsterdam, Amsterdam, the Netherlands; 5 Department of Mental Health and Substance Abuse, World Health Organization, Geneva, Switzerland; 6 Institute for Family Health, Amman, Jordan; 7 Clinical, Neuro and Developmental Psychology, VU University, Amsterdam, The Netherlands; 8 Ministry of Public Health, Beirut, Lebanon; 9 Department of Psychiatry, Faculty of Medicine, Saint Joseph University, Beirut, Lebanon

**Keywords:** Psychological intervention, Randomised controlled trial, Study protocol, Low and middle income countries, Humanitarian emergencies, Armed conflict, Adolescents

## Abstract

**Background:**

There are significant barriers to providing accessible, quality mental health care for young adolescents affected by adversity. In an attempt to overcome this, the World Health Organization (WHO) has developed the Early Adolescent Skills for Emotions (EASE) psychological intervention for young adolescents with internalising problems. EASE is group-based (seven sessions for adolescents, three sessions for their caregivers) and can be delivered by non-specialist providers. This paper outlines the study protocols for two trials of EASE in the Middle East - one in Lebanon and one in Jordan.

**Methods:**

We will conduct two, single-blind, two-arm, individually randomised group treatment trials in Lebanon and Jordan, with at least 445 young adolescents per trial. Adolescents will be screened eligible for the trial if they demonstrate levels of psychological distress indicative of internalizing problems requiring treatment. Participants will be randomly assigned to receive the EASE intervention, or enhanced usual care (one home-visit psychoeducation session). The primary outcome is reduction in overall child-reported psychological distress over time, with 3 months post-treatment as the primary end point. Secondary child-reported outcomes include post-traumatic stress symptoms, depression symptoms, daily functioning, and wellbeing. Secondary caregiver-reported outcomes include parenting style, overall child distress, and caregiver psychological distress. Coping strategy use will be explored as a mediator of treatment effects in Lebanon, and relevant moderators of treatment effects will be explored.

**Discussion:**

These trials will provide the first assessments of the effectiveness of the EASE intervention for use in the Middle East, with important implications for the use of low-intensity, non-specialist interventions for this age range.

**Trial registration:**

Lebanon: ISRCTN75375136. Registered on 11 March 2019.

Jordan: Australia New Zealand Clinical Trials Registry, ACTRN12619000341123. Registered on 5 March 2019 (https://www.anzctr.org.au/)

**Electronic supplementary material:**

The online version of this article (10.1186/s13063-019-3654-3) contains supplementary material, which is available to authorized users.

## Background

There are more than 68 million displaced people in the world today, with over 25 million refugees [1]. More than half of these refugees are children or adolescents, who are at significantly high risk of common mental disorders, such as anxiety and depression [2, 3]. The psychological difficulties experienced by refugee youth is not surprising considering their exposure to war experiences, stressful emigration, acculturation difficulties, and parents’ stress [4]. Furthermore, it is estimated that 85% of the global refugee population are based in low and middle income countries (LMICs) [1], where health systems are often under-resourced to cope with additional vulnerable populations [5]. Host communities in LMICs often face similar ongoing daily stressors associated with living in adversity (poverty, inadequate shelter, high levels of community violence, lack of access to services, etc.), which may have as much or more impact on youth mental health as direct war-related trauma [6, 7].

The recognized need for mental health services in LMICs and humanitarian contexts has led to considerable effort in recent years in assessing the efficacy of applicable mental health programmes. One meta-analysis of individual participant data participant data, comprising data on 3143 children affected by conflict from 11 randomised controlled trials (RCTs), found that focused psychosocial interventions are effective in reducing post-traumatic stress disorder (PTSD) symptoms and in increasing hope, coping, social support, and functioning [8]. However, the psychological programmes in this review required a substantive number of sessions (average of 12 sessions) and there was no effect on depressive and anxiety symptoms. Furthermore, many psychological programmes evaluated and implemented to date are specific to distinct disorders and involve mental health specialists or intensive training of people in order to deliver the programme [9]. These factors have been recognized as major obstacles to scaling up mental health programmes [10]. In response to this situation, many initiatives in LMIC have been trialed in which “task-shifting” approaches have been used that train non-specialist providers to deliver mental health services after brief training; meta-analysis of adults indicates a moderate effect size when programmes are delivered by non-specialist providers [11]. Moreover, there has been a trend for programmes to adopt a transdiagnostic approach insofar as the interventions focus on strategies that address common mechanisms underpinning distress - such as problem solving [12].

In order to provide a scalable solution for mental health care in LMICs, the World Health Organization (WHO) developed a transdiagnostic programme for adults that is brief (five sessions), easy to train non-specialist providers to deliver (8 days of training), and efficacious in reducing psychological distress. Termed Problem Management Plus (PM+) and designed for individuals experiencing emotional disorders, this programme teaches skills in problem solving, arousal reduction, behavioural activation, and accessing social support [13]. Multiple controlled trials have demonstrated the efficacy of PM+ in reducing psychological distress when it is delivered in individual [14, 15] or group [16] format.

There has been a significant need for comparable scalable interventions for young adolescents. In response, the WHO has developed a transdiagnostic programme for 10–14-year-old youths that aims to mitigate symptoms of internalizing disorders, such as depression and anxiety [17]. Termed Early Adolescent Skills for Emotions (EASE), this group-based intervention comprises seven, 90-min sessions that teach the young people skills to enhance psychological coping. On the basis of evidence that children’s mental health is influenced by parents’ mental health and parenting practices [18], and that adjunctive parent sessions can improve the outcomes of psychological interventions for children [19], the EASE intervention includes three sessions for caregivers. EASE has 4 core features that are particularly important for this context: (1) brief in duration; (2) delivered by non-specialist providers; (3) transdiagnostic, addressing depression, anxiety, and distress, and (4) designed for young people and their caregivers living in communities affected by adversity (such as exposure to armed conflict).

This paper presents the study protocol for the initial two RCTs of EASE being conducted in Lebanon and Jordan to determine the effectiveness of the intervention.

## Methods

### Design

**Fig. 1:**
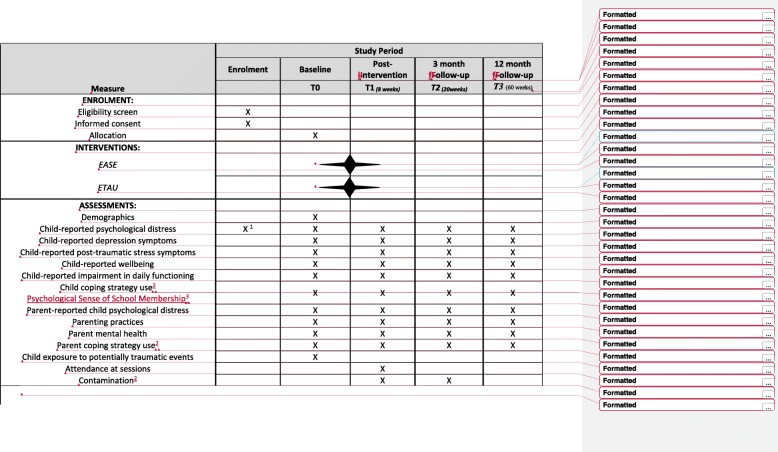
Standard Protocol Items Recommendations for Interventional Trials (SPIRIT): schedule of enrolment, interventions, and assessments for trials of Early Adolescent Skills for Emotions (EASE). ETAU, enhanced treatment as usual. ^1^17-item screener. ^2^Lebanon only. ^3^Jordan only

Two separate two-arm, single-blind, superiority, individually randomised group treatment trials will be conducted, comparing locally adapted EASE interventions to enhanced treatment at usual (ETAU) for reducing symptoms of common mental disorders in (1) Syrian adolescents residing in community settings in Amman, Jordan and (2) adolescents residing in vulnerable regions of Lebanon (including adolescents of Syrian refugee, Lebanese, and other backgrounds). Outcomes on a range of adolescent and caregiver outcomes will be assessed at baseline (T0), post-intervention (T1), 3-month follow up (T2), and 12-month follow up (T3), with the primary outcome point set as T2. The Standard Protocol Items: Recommendations for Interventional Trials (SPIRIT) [20] are outlined in Fig. 1. The completed SPIRIT checklist is available as an Additional file 1.

The Lebanon trial was registered on 11 March 2019, on ISRCTN (10.1186/ISRCTN75375136) and received local ethical approval from St Joseph’s University (ID: USJ – 2017 – 24 bis) and the WHO Ethical Review Committee (Protocol ID: ERC.0003000, 20 March 2018). The Jordan trial was registered on 5 March 2019, on Australia New Zealand Clinical Trials Registry (ACTRN12619000341123; https://www.anzctr.org.au/) and received local ethical approval from the Research Ethics Committee of Al Basheer Hospital and from the WHO Ethical Review Committee (Protocol ID: ERC.0003012, 21 December 2018).

### Aims and hypotheses

The primary aim of both RCTs is to assess the effectiveness of EASE in treating child-reported symptoms of psychological distress at 3-month follow up. The secondary aim is to assess the effectiveness of EASE using other measures of child and caregiver mental health and wellbeing from pre to post intervention, and at 3-month and 12-month follow up. An additional aim is to potentially explore possible treatment moderators (for example, including past traumatic exposures, length of displacement, and attendance at sessions). As an exploratory analysis in the Lebanon site only, we will also assess whether treatment effects are mediated by improvements in the use of coping strategies that align with the proposed EASE mechanisms of action.

We hypothesise that children and caregivers in the EASE arm will show significantly greater improvements on all outcome measures at T1 and T2, and that these gains will be maintained at T3, compared with the ETAU arm. Although the study is not powered to conclusively assess moderation effects, we will conduct exploratory analyses of potential moderators. In Lebanon, we hypothesise that treatment effects will be mediated by an increased caregiver and child use of coping strategies.

### Setting

#### Jordan

The study will be conducted in districts of Amman (Jordan) that have high proportions of residing Syrian refugees. There are currently over 660,000 Syrians registered with United Nations High Commissioner for Refugees (UNHCR) as refugees [21], with the government estimating a total of 1.4 million Syrians residing within Jordan at the current time [22]. The study will be implemented by the Institute for Family Health, a national non-governmental organization (NGO).

#### Lebanon

The study will be conducted in community centres in two governorates of Lebanon-North and Akkar. Lebanon hosts the highest number of refugees per capita, with a current estimate at 1.5 million Syrians, in addition to large numbers of Palestinian refugees (with total national population at 5.9 million) [23]. Lebanon has experienced prolonged internal conflict, external invasions and military assaults, and occupation in recent decades, leading to challenges of limited basic infrastructure, political instability, and struggling economy. Therefore, the Syrian crisis and this rapid and large increase in the population over a short period of time has meant that the ability to meet educational, health, financial, and mental health needs of the population is limited. In areas where Syrian and Palestinian refugees settle, many vulnerable Lebanese families also face similar adversity and lack of services. Most recent estimates indicate only 1.26 psychiatrists and 3.42 psychologists per 100,000 population, with 97% working in the private sector, making mental healthcare largely inaccessible to the most vulnerable [24]. The implementing agency for the study will be War Child Holland, in collaboration with the Ministry of Public Health. War Child in Lebanon has been actively responding to the Syria emergency crisis since early 2012 in the areas of protection, education, and psychosocial support services, providing programmes directly, or through community-based organizations. The study will take place in locations where War Child has active programming.

### Participants

In both sites, participants will be included if they meet the following inclusion criteria: (1) aged between 10 and 14 years; (2) reside with a caregiver who is able to provide consent; (3) is able and willing to commit to attending the weekly EASE sessions; and (4) screens positive for psychological distress during screening. In Jordan, only Syrian refugees will be eligible, while in Lebanon, children of any nationality and background will be eligible.

Participants will be excluded if they meet any of the following criteria: (1) unaccompanied minor; (2) caregiver is not a family member, as they would not be able to provide legal consent; (3) significant cognitive impairment or severe neurological impairments or developmental difficulties as determined by caregiver report during screening, where this would impair their ability to participate in a group programme; (4) imminent risk of suicide; and (5) currently married, due to legal implications regarding consent, and child protection concerns.

In Jordan, where multiple siblings from a family are within the age range to be eligible to take part in the study, only one sibling will be invited to participate in the screening and study. In Lebanon, all eligible siblings will be included in the study, but will be randomised as a single unit to prevent children from the same family being allocated to different intervention arms. This is due to programming policies of the implementing organization (War Child) in Lebanon to include all eligible siblings.

### Recruitment

#### Jordan

Participants will be identified in their homes, through door-to-door screening in the community by assessors hired by the Institute for Family Health. In addition, *snowballing* will be used. Assessors will start sampling at previously determined areas of the community and randomly select a direction in which to screen, selecting households using a population-based interval approach.

After identifying households occupied by Syrian refugees, the assessors will ask to meet with the head of the household. After a brief explanation about the purpose of the survey, they will ask permission to interview one child aged 10–14 years that is currently residing with them. If there are multiple children in this age range residing in the household, the caregiver will be asked which child they would like to take part in the screening.

#### Lebanon

Community engagement and sensitization will involve meetings with relevant community stakeholders to provide them with a basic understanding of the EASE study and to gain their support in the outreach and recruitment process.

Various outreach and recruitment strategies will be used to ensure that eligible children living within the targeted communities are aware of the study and invited to participate. Strategies are expected to include identifying potentially eligible adolescents in active War Child programmes, hosting community awareness sessions, communicating about the study via social media pages frequently used by refugee communities, communicating via other NGO and United Nations partners working in the area, and asking community leaders to ensure all eligible children are given the opportunity to take part. Recruitment will be guided by a script in order to minimize any bias in recruitment amongst the strategies. Adolescents and caregivers meeting inclusion criteria will be registered and invited to attend the screening interview.

### Informed consent and assent

Informed consent from caregivers and assent from children consists of a two-step procedure: (1) to conduct the screening and (2) to take part in the RCT. The second step is only required for participants meeting the inclusion criteria as determined from the screening procedure. Potential participants will be asked to complete a written consent form for each step; for those who are illiterate, witnessed oral consent will be collected, in line with standard practices for trials in such contexts. Following screening, caregivers will be contacted and informed of the results, and for those who screen positive, a time will be scheduled for the T0 assessments and consent for the RCT.

### Assessment of eligibility and screening

After obtaining informed consent and assent, the assessors will record demographic data and administer the screening interview. All screening interviews will be conducted face-to-face by trained research assistants. In Jordan they will take place in the participant’s home. In Lebanon, they will be conducted either in the home, or in a central community centre. In both sites, if the assessors deem the location unsuitable, due to concerns over privacy, the screening will be conducted in an alternative location.

The screening interview with children will consist of the Pediatric Symptom Checklist - 17 (PSC-17-youth report) [25], and an assessment of imminent risk of suicide. The PSC-17 is a brief version of the 35-item Pediatric Symptom Checklist (PSC-35) [26], and measures symptoms of internalising and externalising difficulties in children and adolescents. The recommended cut off score for international use is 15, which is the screening cut off employed in Jordan. The tool has been validated with young adolescents in Lebanon, using semi-structured interviews with psychiatrists as the gold standard (Brown FL, Taha K, Steen F, Aoun M, el Chammay R, Bryant R, Jordans MJD: Validation of Arabic versions of the Child Psychosocial Distress Screener and Pediatric Symptom Checklist for youth living in vulnerable communities in Lebanon, forthcoming). Using these data from the local validation exercise in Lebanon, we determined an optimal cut off of 12 for identifying adolescents meeting 2 criteria: (1) having an internalizing disorder and (2) a clinical indication that treatment is needed. The suicide screening interview will consist of a set of three structured interview questions to identify imminent risk of suicide, as defined by the Mental Health GAP Programme intervention guide [27]. Adolescents at imminent risk of suicide will be referred to specialist support according to the inter-agency standard operating procedures.

The screening interview with caregivers will consist of collecting basic demographic information about the child and family, and assessing four items from an adapted version of the Ten Questions (TQ-10) instrument [28], to assess for significant developmental, neurological, or intellectual impairment that would compromise participation in the programme. Based on this interview, participants will be eligible for the study, and invited to T0 assessments if they (1) score above the specified cut off on the PSC-17 (for Lebanon this will be 12, based on the validation study; for Jordan this will be 15, based on standard cut offs), (2) are not at imminent risk of suicide, and (3) do not have significant impairments.

### Randomisation

Randomisation will occur following completion of the T0 assessment. Randomisation sequences will be computer-generated by an independent staff member who is not involved in study implementation, using a 1:1.6 allocation to EASE or the ETAU group. In Lebanon, to support practical implementation and to ensure adequate numbers in the EASE group programmes, separate randomisation sequences will be created for each location where EASE groups are being held, and within this, separate sequences will be used to create strata for male subjects, female subjects, and sibling pairs. To ensure that the allocation ratio is maintained, blocking will be used with block sizes of 13 (ratio 5:8). Group allocations (EASE or ETAU) will be recorded on pieces of paper, which will be folded and placed inside sealed, numbered, opaque envelopes. The numbered envelopes will be opened in sequence, with the allocation assigned to the corresponding child on registration lists. This will be documented, and the implementing team will be informed of allocations.

### Interventions

#### The Early Adolescent Skills for Emotions (EASE) intervention

EASE is a group psychological intervention, developed by the WHO based on empirically supported strategies recommended by the WHO for emotional disorders in children and young people. The intervention consists of seven 90-min group sessions for adolescents and three 90-min group sessions for their caregivers. Adolescent sessions involve the following strategies: psychoeducation, problem solving, stress management (slow breathing), behavioural activation, and relapse prevention. The caregiver sessions involve psychoeducation, active listening, quality time, praise, caregiver self-care, and relapse prevention. During caregiver sessions, another staff member will be available to provide child care as necessary. Caregiver sessions are scheduled so that the first should occur before the third child session, the second should occur before the fifth child session, and the third should occur before the last child session. More details about the EASE intervention and development have been previously reported [17].

#### Enhanced treatment as usual (ETAU)

Treatment as usual for Syrian refugees in Jordan, and adolescents living in vulnerable communities in Lebanon, usually consists of no intervention. For this study, the comparison group will receive enhanced treatment as usual, which will involve the provision of a single-session, psychoeducation, home visit termed “Psychoeducation for Young Adolescents”. Both the adolescent and caregiver will be invited to the psychoeducation session (of approximately 30 min duration) in which they will receive brief feedback that the youth indicated psychological distress, as well as scripted psychoeducation about (1) self-care strategies and (2) seeking services from local health or community services offering mental health and psychosocial support services. In situations where either the child or caregiver remain concerned about their psychological distress, they will be encouraged to seek support through local community organizations. ETAU participants will not be offered EASE for the duration of their enrolment in the study.

#### Other interventions

Participants will not be prevented from taking part in other interventions during the trial period. In Lebanon, as part of funding arrangements with partners, we will ask participants at T1, T2, and T3 about their health service usage.

## Outcome measures

T1 assessments will be scheduled within 1 week of the final EASE session (i.e. approximately 8 weeks after T0), T2 assessments will be scheduled at 12 weeks following T1 (i.e. approximately 20 weeks after T0), and T3 assessments will be scheduled at 52 weeks following T1 (i.e. approximately 60 weeks after T0).

All instruments have been translated into simple, non-formal Arabic that can be understood by participants in the region (i.e. Syrians, Lebanese, Palestinians, and Jordanians) following recommended procedures for cross-cultural psychology [29]. Steps conducted in Lebanon involved forward translation to Arabic, back translation to English by an independent translator to English, workshops with English-speaking and bilingual team members to review the translations and ensure they retained the original English meaning, cognitive testing with the target population to assess comprehensibility, completeness, relevance, and acceptability, review workshops to adjust as needed, and pilot testing with target populations.

All instruments will be delivered via face-to-face individual interview by trained research assistants, using Kobo electronic data collection software on tablets. Prior to taking part in the study, assessors will receive training on the basics of psychosocial assessments, sensitive interviewing, research ethics, gaining informed consent, study procedures and study instruments, risks of bias in collecting quantitative data, managing participant distress, adverse events reporting procedures, and data management (with role-playing of required skills). Ongoing monitoring of assessors’ competency will be conducted through regular supervision by the research coordinator.

In Jordan, assessments will be conducted in the home. In Lebanon, assessments will either be conducted in the home or in a community centre. Transportation will be provided for participants travelling to the community centre or they will receive reimbursement for any costs incurred in transportation. In the case that participants do not attend a scheduled assessment, three attempts will be made to contact them to schedule a new appointment, via phone calls, home visits, or by contacting alternative contacts provided.

### Primary outcome

The primary outcome is psychological distress as assessed by the PSC-35 youth report [26]. It lists 35 symptoms (including internalising, externalising, somatic, social, and academic difficulties), that are rated for their frequency of occurrence on a 3-point scale ranging from 0 (never) to 2 (often). The total PSC-35 score is obtained by summing the scores of individual items, and ranges from 0 to 70. In a validation exercise in Lebanon, the measure showed high internal consistency (σ = .80), convergent validity, test-retest reliability, and concurrent validity with psychiatric clinical assessments (Brown FL, Taha K, Steen F, Aoun M, el Chammay R, Bryant R, Jordans MJD: Validation of Arabic versions of the Child Psychosocial Distress Screener and Pediatric Symptom Checklist for youth living in vulnerable communities in Lebanon, forthcoming). Since the PSC-35 consists of the 17 items of the PSC-17 plus an additional 18 items, screening scores for children on the PSC-17 items will be used at baseline, and therefore only the additional 18 items administered. We will ensure that there is a maximum of 2 weeks between screening and baseline assessments.

### Secondary outcomes

#### Adolescent-reported outcomes

Symptoms of depression will be measured using the adolescent version of the Patient Health Questionnaire (PHQ-A) [30, 31]. This 9-item checklist asks how often in the past week respondents have experienced symptoms indicative of depressive disorders and is rated on a 4-point scale ranging from 0 (not at all) to 3 (nearly every day). Total scores are calculated by summing responses on all items with a maximum score possible of 27, indicating the highest level of depression symptom severity.

Symptoms of traumatic stress will be measured using the Children’s Revised Impact of Event Scale (CRIES-13) [32]. This 13-item scale measures the psychological and behavioural impact of potentially traumatic events through three subscales exploring intrusion (4-items), avoidance (4-items) and arousal (5-items). The items are rated on a scale of 0 (not at all) to 5 (often) and are added to calculate a severity score, with a maximum possible of 65. Higher scores indicate higher levels of distress consistent with possible post-traumatic stress.

The Impairment of Daily Functioning Questionnaire was developed specifically for these studies, following the process recommended by Bolton [33]. In formative qualitative work in Lebanon, adolescents and caregivers provided input on important daily activities that a child functioning well would be doing. This information was collated into a list of items, and then workshops were held with children where they were asked to group the activities into broader categories and rate the importance of these categories. Nine items were selected based on level of importance and relevance. Adolescents are asked to rate how much impairment they have been experiencing in these activities.

Wellbeing will be measured using the Warwick Edinburgh Mental Wellbeing Scale (WEMWBS) [34]. This measure comprises 14 statements about thoughts and feelings, with respondents asked to indicate which score best describes their experience over the past week on a scale from 1 (none of the time) to 5 (all of the time). Scores across items are summed to arrive at a total between 14 and 70, with higher total scores indicating greater positive mental wellbeing.

In Jordan only, perceived belonging and psychological engagement in school (psychological membership) will be measured using the Psychological Sense of School Membership (PSSM) scale [35]. This multidimensional measure examines membership in school settings, specifically by looking at caring relationships within the school environment, and acceptance and rejection. The scale is composed of 18 items scored on a Likert scale ranging from 1 (not at all true) to 5 (completely true). Final scores are calculated by summing all responses and then dividing by the total number of items to produce an average score ranging from 1 to 5. Higher scores indicate a greater sense of perceived belonging and engagement at school.

In Lebanon only, a child Strategy Use Questionnaire (SUQ) was developed specifically for the Lebanon trial, which consists of 7 items related to the use of coping strategies (identifying emotions, relaxation techniques, behavioural activation, problem solving). Each item is scored on a frequency scale ranging from 0 (never) to 4 (all of the time).

#### Caregiver-reported outcomes

The PSC-35 caregiver report assesses psychosocial impairment and potential emotional and behavioural problems in children [26]. The PSC-35 consists of 35 questions that are scored on a 3-point Likert scale ranging from 0 (never) to 2 (often). The PSC-35 includes three subscales that measure attention and internalizing and externalizing problems. The total PSC-35 score is obtained by summing the scores of individual items and ranges from 0 to 70, with higher scores indicating higher levels of caregiver-perceived psychosocial impairment in children.

Caregiver psychological distress will be measured using the Kessler Psychological Distress Scale (K6) [36]. The K6 consists of six questions pertaining to participants mental health in the previous week, which are scored on a scale from 1 (all of the time) to 5 (none of the time). Total scores range from 6 to 30, and are obtained by summing the individual items. Higher scores indicate higher levels of psychological distress.

The Alabama Parenting Questionnaire-42 (APQ-42) will be used to assess parenting behaviours [37]. The APQ-42 measures 5 parenting constructs: (1) involvement (10 items), (2) supervision and monitoring (10 items), (3) positive parenting (6 items), (4) consistent discipline (6 items), and (5) corporal punishment (3 items). The remaining 7 items assess other disciplinary practices. All items are rated on a 5-point scale ranging from 1 (never) to 5 (always), and scores are calculated for each construct by taking the sum of the relevant items.

In Lebanon only, a caregiver SUQ was also developed, which consists of 8 items related to the use of effective caregiver coping and parenting strategies (identifying emotions in child, comforting child, spending quality time, using praise with children, using harsh discipline, and stress reduction techniques). Each item is scored on a frequency scale ranging from 0 (never) to 4 (all of the time).

In Lebanon, where a caregiver has multiple children in the study, the APQ-42, K6, and caregiver SUQ will only be completed once by the caregiver, while the caregiver-report PSC-35 will be completed separately for each child.

#### Other measures

In order to measure traumatic exposure in children as a demographic characteristic, and possible moderator of treatment effects, we developed a 27-item traumatic events checklist to be delivered to caregivers (at T0 only). The list was developed by pooling items from a range of common trauma checklists (Harvard Trauma Questionnaire [38], University of California Los Angeles Post Traumatic Stress Disorder Revised Inventory [39], Child Posttraumatic Stress Disorder Symptom Scale [40], and Trauma Checklist [41]), and by working with local Lebanese child and adolescent mental health professionals to determine the relevance and acceptability of each item and completeness of the checklist overall, resulting in removal or rephrasing of some items. Each item is scored as “yes” or “no” for occurrence, regardless of when it occurred (in Syria, during migration, or in the current location).

To ensure participant retention in the study we aim to keep detailed address and contact information, and discuss the current location with community members if participants have moved.

### Facilitator selection, training, and supervision

Each EASE intervention will be conducted by two trained facilitators. EASE facilitators will be male and female non-specialist providers recruited from the Institute for Family Health or War Child Holland. They will receive 8 days of training in basic counselling skills, delivery of EASE, group facilitation, and self-care. Additionally, all trained facilitators will be required to complete a practice cycle of the EASE intervention under close supervision. Following training, all facilitators will undergo an assessment of competencies in order to be eligible to implement the intervention. Weekly supervision will be provided by local trainers. These trainers will receive a training-of-trainers, which will include conducting their own EASE intervention groups. They will also receive training in supervisory techniques, in order to ensure protocol adherence. In addition, trainers will receive regular supervision with an EASE master trainer, a clinical psychologist (AM, FB, or MA), to ensure treatment adherence and provide support.

ETAU facilitators will be recruited using the same criteria and process as EASE facilitators. They will receive 3 days of training in delivering the scripted session, basic counselling and communication skills, and self-care. At the end of training, a role-play competency assessment will be conducted. Given the single-session nature of ETAU, facilitators will receive one group supervision session mid-way through implementation of the sessions, and a group debrief and feedback session once all intervention sessions are completed.

### Sample size

The sample size calculation was based on a two-group comparison of the primary outcome at the 3-month follow-up time point. Given that this study is an individually randomised group-treatment trial, it is expected that there will be clustering in the EASE arm due to the group-based delivery of the intervention. Therefore, the sample size should account for this clustering and the potential inflation of outcome variance in the EASE intervention arm. The methods of Moerbeek and Teerenstra (2015) [42] were used, (specifically, eq. 8.14), in order to provide the estimated sample size required in the control arm, given that the following parameters are known: the number of EASE groups (*n*2), the number of members of each group with data at the 3-month follow-up time point (*n*1), the effect size (delta, which is assumed to be the mean difference between arms scaled by the standard deviation in the control arm), the ratio of variance in the EASE arm versus the control arm (theta), and the intracluster correlation coefficient (rho) [43].

In order to obtain estimates for the variance parameters, a small pilot data set from Jordan and Lebanon was used. A conservative estimate of theta (ratio of variances) was 1.1 and of the intraclass correlation was 0.13. Assuming 20 EASE groups of 6 people each at the 3-month follow-up time point, and additionally assuming a 5% two-tailed significance test and 80% power, it is estimated that data from 191 participants in the control arm would need to be available at the 3-month follow-up time point in order to detect an effect size of 0.4. This would correspond to an overall sample size of 311 at 3 months, and an allocation ratio of EASE to ETAU arms of 1:1.6. Allowing for 30% loss to follow up, then the sample size required at enrolment would be approximately 445.

### Statistical analysis

All analyses will be detailed in a statistical analysis plan, which will be signed before unmasking the study data set. Data will be downloaded from the Kobo data collection software and imported into statistical analysis software for data management and analysis. Details of data security and storage can be found in ethical protocols, which are available on request.

To determine comparability between the conditions at baseline, multiple planned comparisons will be conducted for continuous variables and chi squared tests for categorical ones. For hypothesis testing, hierarchical linear modeling (HLM) analysis will be carried out to assess differential change over time in measurement scores between groups. For each outcome, the effects of time of measurement, group, and the group-by-time interaction will be analysed. HLM presumes intent-to-treat analyses, as HLM allows the number of observations to vary between participants and effectively handles missing data. Time (linear and quadratic), treatment condition, and their interaction will be included in the models. Fixed-effects parameters will be tested for intervention conditions, and time of assessments at 95% confidence intervals. The level 1 model will represent within-patient change over time, and the level 2 model will predict variation in within-patient change over time and encompass between-patient variables. Covariates will be added as necessary, including age and gender. Adjustments for clustering at the level of treatment group and sibling (for Lebanon only) will be made during analysis.

Analysis will focus on the primary outcome (PSC-35 youth report) and secondary outcomes of EASE and ETAU, with the main outcome point being the 3-month follow up, relative to baseline. Completers analyses will also be conducted using only the data on participants completing the allocated intervention as planned. In addition to the primary analysis, subsequent analyses will be conducted to explore the roles of potential moderators and mediators on outcomes (independent from primary analyses). Across all analyses, two-tailed tests will be reported with a significance level of *p* < 0.05.

### Implementation and trial management

#### Fidelity of EASE and ETAU

Facilitator pairs will complete a session checklist at each EASE or ETAU session to evaluate treatment fidelity. A sample of 10% of the EASE sessions will be observed by a trained staff member, who will complete a structured observation form to score which elements of the programme have been carried out by the facilitator, and to what quality. Similarly, a sample of 10% of ETAU sessions will be observed and a checklist completed.

The competency of the EASE facilitators will be tested before and after participation in training, using a modified version of the Enhancing Assessment of Common Therapeutic factors (ENACT) rating scale for training and supervision [44]. The ENACT scale is an 18-item assessment for common factors in psychological treatments, including task-sharing initiatives with non-specialists across cultural settings. We will utilize 5 of the items for this trial. Four competency items will also be assessed during each session observation.

#### Blinding

Participants and implementation staff will not be blind to participant allocation. The research assistant team will remain blind to the intervention allocation of children throughout the trial, and will operate independently from the intervention facilitators. All staff have been trained and supervised in the importance of maintaining blinding, and at no time will intentional unblinding of the research assistants be required. Prior to conducting each T1, T2, and T3 assessment, instructions will be given by research assistants to all participants about the importance of not revealing their allocation. In the case that the allocation is revealed, research assistants will be instructed to inform the research coordinator immediately and another research assistant will complete the assessment with that participant. At the end of each T1, T2, and T3 assessment, research assistants will provide a guess as to which treatment the participant received - if blinding was maintained, these guesses should be no better than chance.

#### Contamination

In Lebanon, in order to assess the extent of contamination across EASE and ETAU arms, participants in both the EASE and ETAU arms will be asked several structured questions at T1 and T2 about the extent to which they shared information and materials about the treatment received with others in the community, and whether they have heard about the other treatment and materials from others. This information will be used descriptively to determine contamination.

#### Trial monitoring

In each site, a trial management committee consisting of principal investigators, co-investigators, and research coordinators will regularly monitor the implementation of study procedures. All adverse events (AEs) (e.g. injuries on the way to treatment, increase in distress) and serious adverse events (SAEs) (e.g. suicide attempts, serious violence) will be recorded by the research team and reported to a site-specific Data Safety Management Board (DSMB). Meetings will be facilitated by the study coordinator, but the board will consist of three or more local professionals, external to the study, but with experience in similar research. The PI in each site will be responsible for reporting (S)AEs to the board, and also to relevant ethics committees. The chair or a nominated person from the advisory board will review SAEs within 48 h and the advisory board will review all AEs once a month and where necessary to determine any appropriate action in respect of ongoing trial conduct. Information is included on the informed consent form to inform participants that the field coordinator or another clinician other than their therapist are available to them if they are upset by this study. If necessary, appropriate action will be taken with respect to individual participants or conduct of the trial (such as referral to specialised care, installing extra assessment points for monitoring participants, or discontinuation). No interim analyses are planned. The local project coordinator is responsible for ensuring timely follow up of any (S)AEs, and will inform the participants and DSMB if any data indicate that the disadvantages of participation may be significantly greater than expected.

## Discussion

EASE has been developed with the aim of reducing the treatment gap for young adolescents living in adversity and affected by psychological distress. The aim is to provide a brief, readily scalable intervention that can be delivered by trained and supervised non-specialists in low-resource settings such as humanitarian settings and LMICs, and cover a range of psychological distress presentations. The trials outlined in this protocol are the first trials to assess the effectiveness of EASE when implemented in challenging settings in Jordan and Lebanon, with adolescents living in adversity and experiencing psychological distress. If effectiveness is demonstrated, EASE may be scaled up in these contexts, and adapted and scaled out to adolescents experiencing adversity in other settings. Assuming positive effects are identified, the EASE manual and accompanying materials will be published by WHO and will be made freely and publicly available on their website.

## Trial status

Recruitment for the Jordan trial is planned to commence in March 2019 and continue until August 2019. Recruitment for the Lebanon trial is planned to commence from June 2019 and continue until August 2019.

## Additional file

Additional file 1: SPIRIT 2013 checklist: Recommended items to address in a clinical trial protocol and related documents. (DOCX 45 kb)

## Data Availability

Data and materials will be shared upon request to Richard Bryant, University of New South Wales, Sydney, Australia (Jordan), and Mark Jordans, War Child Holland (Lebanon).

## Abbreviations

AE: Adverse event
APQ-42: Alabama Parenting Questionnaire 42
CRIES- 13: Child Revised Impact of Events Scale 13
DSMB: Data Safety Management Board
EASE: Early Adolescent Skills for Emotions
ENACT: Enhancing Assessment of Common Therapeutic factors
ETAU: Enhanced treatment as usual
HLM: Hierarchical linear modelling
K6: Kessler Psychological Distress Scale 6
LMIC: Low and middle income country
NGO: Non-governmental organisation
PHQ-A: Patient Health Questionnaire for Adolescents
PM+-: Problem Management Plus
PSC-17: Pediatric Symptom Checklist 17
PSC-35: Pediatric Symptom Checklist 35
PSSM: Psychological Sense of School Membership
PTSD: Post-traumatic stress disorder
RCT: Randomised controlled trials
SAE: Serious adverse event
SUQ: Strategy Use Questionnaire
TQ-10: Ten questions
WEMWBS: Warwick Edinburgh Mental Wellbeing Scale
WHO: World Health Organization
