# Correction to: Early Adolescent Skills for Emotions (EASE) intervention for the treatment of psychological distress in adolescents: study protocol for randomised controlled trials in Lebanon and Jordan

**DOI:** 10.1186/s13063-019-3717-5

**Published:** 2019-10-29

**Authors:** Felicity L. Brown, Frederik Steen, Karine Taha, May Aoun, Richard A. Bryant, Mark J. D. Jordans, Aiysha Malik, Mark van Ommeren, Adnan Abualhaija, Ibrahim Said Aqel, Maha Ghatasheh, Rand Habashneh, Marit Sijbrandij, Rabih El Chammay, Sarah Watts, Aemal Akhtar, on behalf of the STRENGTHS-consortium

**Affiliations:** 1 Research and Development Department, War Child Holland, Amsterdam, The Netherlands; 2 War Child Holland Lebanon Office, Beirut, Lebanon; 3 School of Psychology, University of New South Wales, Sydney, Australia; 4 Amsterdam Institute of Social Science Research, University of Amsterdam, Amsterdam, the Netherlands; 5 Department of Mental Health and Substance Abuse, World Health Organization, Geneva, Switzerland; 6 Institute for Family Health, Amman, Jordan; 7 Clinical, Neuro and Developmental Psychology, VU University, Amsterdam, The Netherlands; 8 Ministry of Public Health, Beirut, Lebanon; 9 Department of Psychiatry, Faculty of Medicine, Saint Joseph University, Beirut, Lebanon

**Correction to: Trials (2019) 20:545**

**https://doi.org/10.1186/s13063-019-3654-3**

Following publication of the original article [1], we have been notified that Fig. 1 was published with the track changes. In this Correction the incorrect and correct Fig. 1 are shown.

**Figure:**
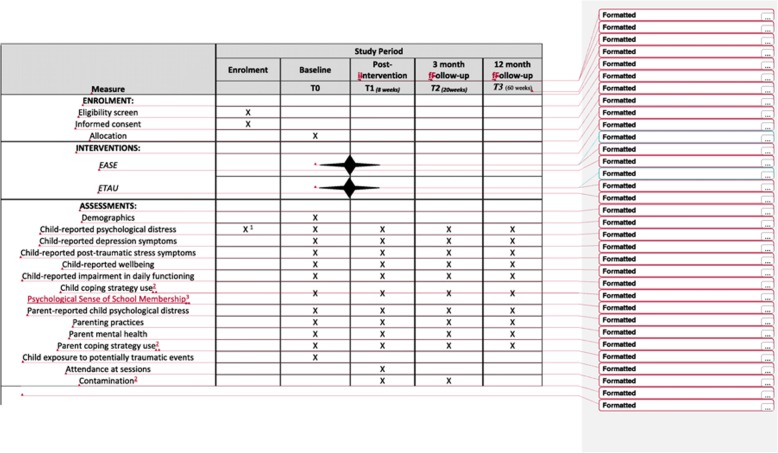


**Fig. 2:**
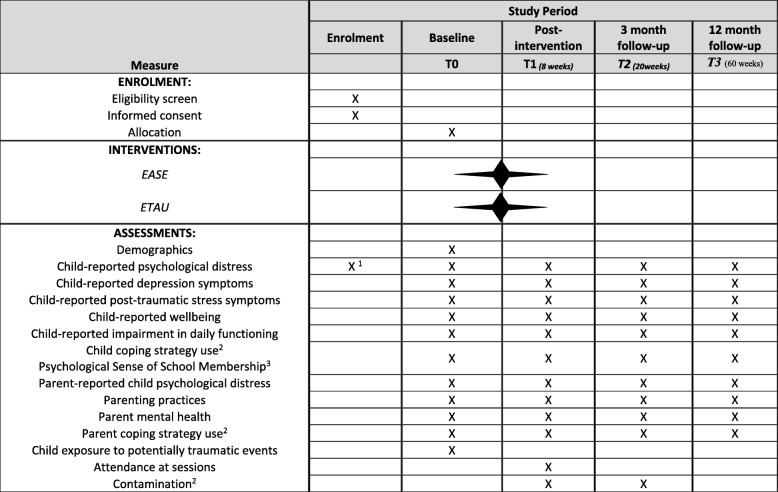
Standard Protocol Items Recommendations for Interventional Trials (SPIRIT): Schedule of enrolment, interventions, and assessments for trials of EASE. ^1^ 17-item screener. ^2^Lebanon only. ^3^ Jordan only

The original article has been corrected.
